# Burnout among physicians and caregivers in oncology: the Moroccan experience

**DOI:** 10.3332/ecancer.2022.1473

**Published:** 2022-11-17

**Authors:** Yassine Benhaddouch, Samia Khalfi, Soumaya Benmaamar, Chaymae Marzouki, Nour El Houda Fares, Mohamed Sbai, Kaoutar Soussy, Bouthaina Assenhaji, Hajar Filali, Youssef Aghlallou, Amine Bout, Chadia Aarab, Rachid Aalouane, Fatimazahra Farhane, Zenab Alami, Touria Bouhafa

**Affiliations:** 1 Department of Psychiatry-Addictology, CHU Hassan II of Fez, Fez, Morocco; 2 Department of Radiotherapy and Brachytherapy, CHU Hassan II of Fez, Fez, Morocco; 3 Department of Epidemiology, Faculty of Medicine and Pharmacy of Fez, Fez, Morocco; 4 Cancer Research Institute of Fez, Fez, Morocco

**Keywords:** psycho-oncology, professional burnout, depression, Maslach Burnout Inventory, Generalised Anxiety Disorder-7 scale, Patient Health Questionnaire, oncology caregiver

## Abstract

**Purpose:**

Caregivers in radiation oncology are exposed to a high risk of burnout which sometimes causes serious consequences for their health, which can in turn affect patient care. In this study, we investigated the prevalence of burnout and its psychological impact on health professionals and determined the factors that predispose to the risk of burnout.

**Methods and materials:**

A cross-sectional survey was conducted with descriptive and analytical purposes among the different teams within the oncology and radiotherapy departments in different hospitals (CHU and regional hospitals) in Morocco, through an online self-questionnaire composed of sociodemographic data, professional data, working conditions, an assessment of interfering factors, the Maslach Burnout Inventory (MBI), the Generalised Anxiety Disorder-7 (GAD-7) scale and the Patient Health Questionnaire-9 (PHQ-9) scale.

**Results:**

One hundred and eighteen caregivers participated in this evaluation. 62.7% were physicians, 75.4% worked in university hospitals and 53.4% were in radiotherapy departments. Analysis of the three dimensions of the Maslach Burnout Inventory - Human Services Survey (MBI-HSS) score found high scores in the dimensions of emotional exhaustion (81.4%) and depersonalisation (79.7%), and low scores in decreased personal accomplishment (46.6%). The evaluation of the impacts of burnout in the latter found a mean of the Patient Health Questionnaire-9 (PHQ-9) at 12.45 + 7.84 and the mean of the GAD-7 at 9.73 + 5.98.

**Conclusion:**

Our results are in line with those found in the literature, hence the need for screening and active prevention of burnout among radiation oncology caregivers.

## Introduction

As the second leading cause of death in the world, cancer claims approximately 10 million lives per year. Nearly one in every six deaths is due to cancer worldwide [1]. As a result, more and more healthcare professionals are taking care of cancer patients. These professionals are likely to face the usual tensions already existing in caregiver–client relationships, but also to face tensions more specific to cancer illness, such as the confrontation with suffering and death [2].

Burnout is found in professions faced with failure and feelings of powerlessness, particularly in the medical professions and in hospital departments with high mortality rates, such as oncology departments. Freudenberger was the first to define burnout [3]. He mentions feelings of helplessness and guilt, sadness, as well as boredom and disinterest. This would reflect the state of exhaustion of staff ‘consumed’ by a job that is relationally demanding, repetitive and ultimately appears to have no way out. Maslach and Jackson [4] emphasise ‘the development of a pejorative vision of oneself and negative attitudes towards work, as well as a loss of interest and feeling towards clients’. Burnout would be a failure to adapt to the chronic emotional stress of work [5].

According to Maslach, the burnout syndrome is a sequence of three stages. The first stage is emotional exhaustion (EE), which reflects the degree to which a person feels emotionally overworked and exhausted by his or her work. The second stage is depersonalisation (DE), which represents the degree to which a person has developed feelings of indifference and cynical attitudes toward the recipients of the care, treatment, instruction, or services they provide to others in their work. The third stage is personal accomplishment (PA), which is the degree to which a person experiences a sense of competence and achievement in their work [5].

Although many professionals can experience burnout, it is more common among healthcare professionals [5] and especially among professionals in oncology and critical care [7, 8]. Several studies conducted worldwide in recent years have assessed the prevalence and impact of burnout among healthcare professionals in oncology departments, and they find that oncologists are more emotionally and psychologically demanding than other specialities. In a study conducted in the United States [9] in 2017 showed a significant level of burnout among healthcare professionals with a prevalence increased over the years estimated at 45.6%. A systematic review of 182 studies from 45 countries showed a prevalence of burnout of up to 80.5% [10]. A recent survey conducted in 2020 among health professionals in the Middle East and North Africa (MENA) region showed a prevalence of burnout at 68% with significantly high scores of EE and DE (35% and 57%, respectively) with lower scores of professional accomplishments (49%) [11].

Burnout often causes symptoms that can negatively affect the quality of life of healthcare professionals. These symptoms include anxiety, depression, sleep disturbances and addictive disorders [12]. Studies have shown significant associations between burnout and reductions in work performance and stress-related health problems [13].

To the authors’ knowledge, there are few studies that have addressed the problem of burnout among health professionals in oncology departments in the MENA region [11, 14].

In Morocco, little is known today about the distress faced by cancer caregivers and their consequences. Our work represents the largest study assessing burnout among professionals in oncology services in different hospitals in Morocco, and aims to study the psychological impact of burnout on these professionals in order to determine the factors predisposing to burnout.

## Methods

### Purpose of this work

Through this study, we want to answer a certain number of questions, such as: What are the socio-demographic factors that increase the risk of burnout? Does the risk of burnout change according to professional status or workplace (radiotherapy or medical oncology department, university hospital or regional hospital)? Does workload and working conditions increase the risk of burnout? Does the risk of burnout increase with certain interfering factors (chronic illness, family factors, psychiatric or substance use history)?

### Sample size determination

The sample size was calculated by using the proportion formula with 95% confidence level, 5% margin of error and proportion of burnout among oncologists. Proportion, which is 68%, was taken from a study conducted on burnout among oncologists from North Africa and Middle East (BOMENA Study) [11].


$$N=\frac{P(1-P)}{I^{2}}(\mathrm{Za}{/2)}^{2}$$

Where *N* is the sample size, Za/2 = 1*.*96 (standardised normal distribution curve value for the 95% confidence interval), P = 0. 68 (proportion of) and I = 0*.*03 (precision).

*N* = 335

Since we have a limited population of oncologists: we used the following correction:


$$N_{2}=\frac{N}{1\frac{N}{Q}}(\mathrm{Za}{/2)}^{2}$$

Q: number of oncologists in regional hospitals and university centres = 180

*N*_2_= 118

### Study design and data collection

A cross-sectional survey was conducted with descriptive and analytical purposes among the different teams within the medical oncology and radiation oncology departments in eight hospitals (University hospitals and regional hospitals) in Morocco, through an online self-questionnaire. All participants individually completed this questionnaire in two parts. The first part consisted of socio-demographic data (age, gender, marital status, presence of children, housing), professional data (clinical status (including resident physicians or specialists and paramedical function including nurses, technicians and orderlies), place of work, department of practice, number of years of practice and number of on-call hours per month), working conditions (number of staff, workload, presence of protective equipment, presence of therapeutic equipment and means, organisation of work, communication within the team, support and recognition of the team), an evaluation of interfering factors (presence of chronic illness, presence of a family member being followed for a chronic pathology or cancer, presence of a psychiatric disorder, presence of an addiction).

### Study instrument

The second part dealt with an evaluation of burnout and its impacts. We used the three scales to screen for burnout, depression and anxiety disorders in healthcare professionals.

Maslach Burnout Inventory - Human Services Survey (MBI-HSS) scale used to evaluate burnout in the medical field. It includes 22 items and each question corresponds to seven answers ranging from 0 to 6 meaning ‘never’ to ‘every day’. The MBI tool provides three subscales that, respectively, measure the following three dimensions: ‘Emotional Exhaustion’ (EE) is the feeling of care professionals of being drained and emotionally undergoing work. ‘Depersonalization’ (DE) is the staff’s emotional distancing from the people in their care. This is not a dissociative disorder that damages self-awareness in the sense understood by the Diagnostic and Statistical Manual of Mental Disorders, 4^th^ Edition (DSM IV) [15], but a dimension that reflects ‘the development of impersonal, detached, negative, cynical attitudes toward the people these care professionals care for’ [16]. Reduced ‘personal accomplishment’ (PA) at work, which reflects the disengagement and deep demotivation of care professionals with respect to work. Questions 1, 2, 3, 6, 8, 13, 14, 16 and 20 assess the dimension of EE; scores 5, 10, 11, 15 and 22 assess the dimension of DE and scores 4, 7, 9, 12, 17, 18, 19, 21 assess the dimension of PA. Burnout appears for high scores in the EE and ED dimensions and for low scores in PA.

Generalised Anxiety Disorder-7 (GAD-7) scale is a relevant tool for the screening of generalised anxiety disorder in patients. It is a self-administered questionnaire with seven items and each question corresponds to four answers ranging from 0 to 3 [17].

Patient Health Questionnaire-9 (PHQ-9) is a tool for the diagnosis and measurement of the severity of depressive symptoms. It is a self-administered questionnaire adapted to the DSM-IV criteria, composed of nine items and each question corresponds to four answers [18]. A score higher than 20 corresponds to a severe depression.

The French version was used because these scales are not validated in Moroccan Arabic dialectal.

### Statistical analysis

The questionnaire data was processed on Excel software and then analysed by R software. The categorical variables were represented in percentages. The continuous (quantitative) variables were represented in means ± standard deviation. The analysis of the factors associated with the different scales used the following tests: The Chi-2 test for the comparison of percentages, the *t*-test and Analysis of variance (ANOVA) for the comparison of means. Pearson’s correlation was used to study the association between the quantitative variables. The value *p* < 0.05 was considered statistically significant.

## Results

There were 122 participants in this survey, but only 118 willing caregivers agreed to complete all items.

### Descriptive results

A total of 118 health care workers participated in this survey, whose average age was 33.51 ± 6.86 years, 64.4% were female, 50% were married, 55.9% had no children, 50% lived with a spouse and/or child, 79.7% had no personal history of chronic disease, 73.7% had relatives being monitored for chronic disease or cancer and 75.4% had no addictions.

Among the participants, 62.7% were physicians, 75.4% worked in university hospitals and 53.4% were in radiation oncology departments (Table 1).

Most of the participants (60.2%) felt that the number of staff was sufficient, despite the majority feeling the high workload (65.3%). Also, the protection means were insufficient in 58.5% and the equipment and therapeutic means were sufficient in 55.1%.

Three quarter of the participants thought the organisation of work was insufficient, the communication within the team was sufficient (72,9%), but the team support and recognition was insufficient (50.8%) (Table 2).

In this survey, it was found that the average years of practice for cancer service staff was 5.86 + 4.90 years and the number of shifts per month was 3.85 + 3.68, because oncology services started recently in Morocco.

Analysis of the three dimensions of the MBI-HSS score found high scores in the EE and DE dimensions; however, low scores in diminished PA (Table 3), reflecting the presence of burnout among cancer service professionals.

The assessment of the impacts of burnout in the latter found a mean PHQ-9 of 12.45 + 7.84 and a mean GAD-7 of 9.73 + 5.98.

## Analytical results

We conducted a univariate study to determine the predictive risk factors for the occurrence of burnout, anxiety and depressive disorders in healthcare professionals in cancerology departments. To do this, we studied the associations between the three scales: MBI-HSS, PHQ-9 and GAD-7, and the other variables of interest in search of associations.

**Overall results of the MBI-HSS:** For statistical purposes (small number of participants = 118 cases), two levels of severity were grouped into the three dimensions of the MBI scale (low or moderate and severe).

Predictive factors for high scores of the different components of burnout:

Physicians were found to have higher scores on the EE dimension (87.8% versus 70.5%; *p* = 0.01), DE dimension (87.8% versus 65.9%; *p* = 0.004) and lower PA (78.4% versus 65.0%; *p* = 0.13) than allied health professionals.

Caregivers who felt that communication within the team was sufficient had a high EE score (86%; *p* = 0.03), as well as reduced PA (80.2%; *p* = 0.008).

Professionals in the medical oncology department had a higher EE score than professionals in the radiation oncology department (89.1% versus 74.6%; *p* = 0.04).

A high number of shifts per month was present among professionals with a high EE score (5.66 ± 4.49; *p* = 0.01), a high DE score (6.04 ± 4.22; *p* = 0.002) and a reduced PA (5.82 ± 3.83; *p* = 0.001).

In addition, a reduced score of PA was found among caregivers who considered that the protective measures were insufficient (81.2%; *p* = 0.03) and that the work organisation was insufficient (84.1%; *p* = 0.0001).

A high score on the EE dimension was also found among caregivers who had a loved one with a chronic disease or cancer (85.1%; *p* = 0.08) and among those who worked in a regional hospital (93.1%; *p* = 0.06). A high score on the DE dimension was present in men compared to women (88.1% versus 75.0%; *p* = 0.09) and in caregivers with a history of psychiatric follow-up (90.3%; *p* = 0.08) and a lower PA score in caregivers with a higher age (34.17 + 5.98; *p* = 0.08) (Table 3). These high scores were not statistically significant.

### Results of the PHD-9 and GAD-7 scales

#### Predictive factors for depression and anxiety:

Caregivers with a history of psychiatric follow-up had high depression (16.87 + 7.96; *p* = 0.0001) and anxiety (13.03 + 5.57; *p* = 0.0001) scores.

There was a statistically significant association between sufficient workload and depression (13.96 + 7.84; *p* = 0.004) and anxiety (10.80 + 5.73; *p* = 0.007).

A significant association was present between depression and marital status (*p* = 0.03). Divorcees had higher depression scores, followed by singles versus married (16.55 + 8.06 versus 12.70 + 8.63 versus 11.03 + 6.80).

Higher levels of anxiety were observed among caregivers who did not have children (*p* = 0.029), among physicians (*p* = 0.07) and among those who felt that communication within the team was inadequate (*p* = 0.07) (Table 5).

## Discussion

In recent years, some studies have focused on burnout among healthcare professionals, particularly in oncology services, since they are confronted daily with failure, stress and feelings of powerlessness. Nevertheless, few studies have evaluated the frequency, associated factors and impacts of burnout in these professionals.

In Morocco, the link between stress and the mental health of health professionals remains taboo. Very few epidemiological studies have been conducted, unlike in Anglo-Saxon countries. However, work overload (rhythm and number of consultations) and its impact on private life, temporal and organisational constraints (on-call duty), the solitude of medical practice (announcement of bad news), confrontation with the physical and psychological suffering of patients, the growing complexity of scientific bases and medical techniques, relational difficulties with patients and families, with increasing judicialisation, administrative problems and ‘confraternal’ or institutional tensions all contribute to the stress and exhaustion of physicians, particularly in hospitals [19].

The average age of the participants in our survey was very young at 33.51 years, due to the fact that it is a developing discipline in our Moroccan context. This result is comparable to the study conducted in the MENA region, where the majority of participants were younger than 45 years [11]. In an American study, the average age of the participants was 52 years and only 5.8% were under 40 years [20].

Several factors, including locally applied access-to-care strategies, the number of oncology care staff, bureaucratic and administrative tasks, disease patterns and population health status may explain the prevalence of burnout in different countries.

Although several surveys have shown that gender is not a significant factor in burnout [21, 22], the study by Ozyurt *et al* [21] reported that female gender was associated with a high risk of developing burnout. In our work, we find similar results to the study conducted in the MENA region, where female oncologists had a higher prevalence of EE and low PA than men [11]. This result could be explained by lack of mentorship, lack of trust from patients and being a working mother dealing with the needs of her family [24, 25].

In the present work, we find an increase in the rate of burnout among physicians with significantly elevated levels of EE and DE, and also elevated levels of EE among professionals in oncology departments and among those practicing in regional hospitals.

Our results are consistent with those of previous studies in which physicians were more exposed to high rates of burnout. In a US study conducted between 2011 and 2015 [26], an increase in the rate of burnout over time was observed among physicians. Another survey of 340 oncologists in France found that 44% of professionals were burnt out and wanted to stop working in the medical sector [27]. In the MENA study [11], oncologists in the northern regions of Africa had an estimated 74% high level of burnout. Also, Allegra *et al* [26] found that among 1,740 oncology physicians, 69% had symptoms of EE, 78% had symptoms of DE and 50% had reduced professional achievement.

Eelen *et al* [27], in their study, showed that physicians in medical oncology departments had significantly high levels of EE and DE. These results are similar to those of our study and can be explained by the fact that medical oncologists mostly treat patients with metastases or at the end of life with a higher mortality rate compared to radiation-oncologists who mostly treat curable cancers. In the study conducted in the MENA region [11], we find that almost 95% of the participating professionals agreed that oncology is more emotionally and psychologically demanding than other specialities.

In our work, a high number of on-calls per month was associated with a higher level of burnout among healthcare professionals. This figure extended to the subscales (EE, DE and PA). A meta-analysis [30] found that high workload and on-call time were associated with an increased chance of developing burnout. Although the study conducted in the MENA region [11] found that professionals who valued interpersonal relationships and communication in oncology were 50% less likely to have burnout than those who did not. In our work, 72.9% of physicians who felt that team communication was sufficient had significantly high levels of EE and low levels of PA. In our study, 58.5% felt that protective equipment was insufficient and 74.6% felt that work organisation was insufficient. This lack of equipment and organisation was significantly associated with a very low level of PA. In a study by Batra K *et al* [38], it was shown that the lack of work organisation and equipment were risk factors for medical doctor’s mental health. Another study showed that among senior physicians in radiation oncology, organisational factors as well as the work environment and lack of continuing professional development were correlated with an increased risk of burnout [32]. Consequences of burnout include low self-esteem, isolation, depression, anxiety, job dissatisfaction, early retirement or resignation [20], which affect the services provided, resulting in reduced quality of care and patient safety [33, 34]. In the literature, studies of physicians in oncology departments in different countries have shown rates of depressive symptoms ranging from 8.8% to 28.1% [35–37].

In a Chinese study [39], a prevalence of 25.6% of anxiety symptoms was found in a large sample of oncology physicians. We found similar results when assessing depression and anxiety in oncology professionals with scores ranging from moderate to moderately severe. In the literature, workload, low involvement in decision making, pressure to perform and work–life balance challenges have been considered as factors responsible for the occurrence of depression and anxiety [40]. In our work, increased workload is a risk factor for developing depression and anxiety. Our study also found a significant association between caregivers’ psychiatric history and the occurrence of depression and anxiety, and having children and being married are protective factors against depression and anxiety.

### Clinical implications

Our cross-sectional study, conducted among oncology caregivers, assessed burnout and its psychological impact among these caregivers and detected risk factors for this impact, while using an anonymous self-questionnaire including different psychometric scales to assess burnout, depression and anxiety. This study brought out interesting descriptive and analytical results. Nevertheless, the results of this work constitute the basis for further work and improvement for a much more in-depth and long-term study.

### Study limitations

This study is limited by the small number of participants since participation in the study was voluntary, the scales used are not validated in dialectal Arabic; however, the French version used is adapted since Moroccan health professionals pursue medical studies in French which makes the use of the French version of the score legitimate. Moreover, we have the social desirability bias, we assessed the level of burnout only at a given time, so we have no idea of the psychological state of the participants at the time they answered the questionnaire, and finally we find rare similar studies in our country and neighbouring countries which limits us to compare our results.

## Conclusion

To the authors’ knowledge, our study is the largest study evaluating burnout among healthcare professionals in oncology departments in Morocco. We have included a large sample and we have identified modifiable risk factors for burnout in oncology health professionals in order to establish new treatment strategies focused on the individual and on the organisation of work.

## Conflicts of interest

The authors declare no conflicts of interest.

## Funding

Moroccan research institute IRC. Address: Route sidi Harazem, Rte du parc shore, Fez 30070. Research funded by research cancer institute IRC. www.irc.ma project number 709/Aamp 2019.

## Figures and Tables

**Table 1.** Description of sociodemographic and professional data.

Variables	Number of employees (in percent)
Gender • Female • Male	76 (64.4%) 42 (35.6%)
Marital status • Single • Married • Divorced or widowed	41 (34.7%) 59 (50%) 18 (15.3%)
Presence of children • No children • Yes	66 (55.9%) 52 (44.1%)
Personal history of chronic disease • No • Yes	94 (79.7%) 24 (20.3%)
Family history of chronic disease • No • Yes	31 (26.3%) 87 (73.7%)
History of psychiatric follow-up • No (past or present) • Yes (past or present)	87 (73.7%) 31 (26.3%)
Notion of addictions • No • Yes	89 (75.4%) 29 (24.6%)
Housing • Spouse and/or children • In collocation • Parents • Alone	59 (50%) 9 (7.6%) 31 (26.3%) 19 (16.1%)
Function • Medical (residents and specialists) • Paramedical (nurses, technicians...)	74 (62.7%) 44 (37.3%)
Place of work • University hospital • Regional hospital	89 (75.4%) 29 (24.6%)
Department of practice • Medical oncology • Radiation oncology	55 (46.6%) 63 (53.4%)

**Table 2.** Description of working conditions.

Team size • Insufficient • Sufficient	47 (39.8%) 71 (60.2%)
Workload • Low or medium • High	41 (34.7%) 77 (65.3%)
Presence of protection means • Insufficient • Sufficient	69 (58.5%) 49 (41.5%)
Equipment and therapeutic means • Insufficient • Sufficient	53 (44.9%) 65 (55.1%)
Organisation of the work • Insufficient • Sufficient	88 (74.6%) 30 (25.4%)
Communication within the team • Insufficient • Sufficient	32 (27.1%) 86 (72.9%)
Team support and recognition • Insufficient • Sufficient	60 (50.8%) 58 (49.2%)

**Table 3.** Predictive factors of burnout in health care professionals.

MBI	EE			DE			PA		
	Low or moderate	High	*p*	Low or moderate	High	*p*	Low or moderate	High	*p*
Age	NS			NS			34.17 ± 5.98	31.67 ± 8.74	**0.08**
Gender • Female • Male	NS			25.0% 11.9%	75.0% 88.1%	**0.09**	NS		
Function • Medical • Paramedical	12.2% 29.5%	87.8% 70.5%	**0.01**	12.2% 34.1%	87.8% 65.9%	**0.004**	78.4% 65.0%	21.6% 34.1%	**0.13**
Presence of protection means • Insufficient • Sufficient	NS			NS			81.2% 63.3%	18.8% 36.7%	**0.03**
Organisation of the work • Insufficient • Sufficient	NS			NS			84.1% 43.3%	15.9% 56.7%	**0.0001**
Communication within the team • Insufficient • Sufficient	31.3% 14.0%	68.8% 86.0%	**0.03**	NS			56.3% 80.2%	43.8% 19.8%	**0.008**
Place of work • University hospital • Regional hospital	22.5% 6.9%	77.5% 93.1%	**0.06**	NS			NS		
Department of practice • Medical oncology • Radiation oncology	10.9% 25.4%	89.1% 74.6%	**0.04**	NS			NS		
Number of shifts per month	3.44 ± 3.36	5.66 ± 4.49	**0.01**	3.35 ± 3.37	6.04 ± 4.22	**0.002**	5.82 ± 3.83	3.16 ± 3.39	**0.001**

NS, Non-specific

**Table 4.** Description of the three dimensions of burnout by MBI-HSS.

MBI	Low	Moderate	High
EE (in percent)	12 (10.2%)	10 (8.5%)	90 (81.4%)
DE (in percent)	11 (9.3%)	13 (11%)	94 (79.7%)
PA (in percent)	55 (46.6%)	32 (27.1%)	31 (26.3%)

**Table 5.** Predictive factors of depression and anxiety.

	PHQ-9		GAD-7	
	Average score ± standard deviation	*p*	Average score ± standard deviation	*p*
Marital status • Single • Married • Divorced or widowed	12.70 ± 8.63 11.03 ± 6.80 16.55 ± 8.06	**0.03**	10.53 ± 7.14 8.57 ± 4,98 11.72 ± 5.56	**0.08**
Presence of children • No children • Yes	NS		9.73 ± 5.28 10.80 ± 6.31	**0.029**
History of psychiatric follow-up • No • Yes (past or present)	10.88 ±7.21 16.87 ±7.96	**0.0001**	8.56 ± 5.70 13.03 ± 5.57	**0.0001**
Function • Medical • Paramedical	NS		10.50 ± 5.96 8.45 ± 5.86	**0.07**
Workload • Low or medium • High	9.63 ± 7.10 13.96 ± 7.84	**0.004**	7.73 ± 5.99 10.80 ± 5.73	**0.007**
Team support and recognition • Insufficient • Sufficient	NS		10.70 ± 6.39 8.74 ± 5.40	**0.07**

NS, Non-specific
